# On Ulcers of the Cornea

**Published:** 1845-09

**Authors:** W. White Cooper

**Affiliations:** Senior Surgeon to the North London Ophthalmic Institution, &c.


*On Ulcers of the Cornea.* By W. White Cooper, Esq., F. R. C. S., Senior Surgeon to the North London Ophthalmic Institution, &c.—Ulceration of the cornea is one of the diseases of the eye most common amongst the lower classes, and it is by no means unfrequent in the higher orders. Some forms are exceedingly intractable, especially in children, and the disease will go on, week after week, and month after month, sometimes better, at other times worse, until the patience of the surgeon is worn out, and the friends of the child are in despair. As I have had considerable experience in the treatment of these affections, I venture to offer a few practical remarks, hoping that they may not be altogether useless.

Ulcers are very commonly attendant upon strumous ophthalmia, of which a characteristic symptom is intolerance of light, and it is often extremely difficult to obtain a satisfactory view of the eye. If the patient be a child, he is sure to resist any attempt to open the eye; he will struggle, and fight, and scream, most furiously, and if we endeavour to raise the lid, the chances are, that it becomes everted, and that the cornea will be so completely concealed by it, that its surface cannot be seen. All this, too, produces congestion of the eye, so that even if we do obtain a view, a fair opinion cannot be formed as to the state of matters, and it must not be forgotten that if the ulcer be deep, serious mischief may arise if much violence is made use of in the examination. There are two modes which I have found to obviate all these inconveniences. Either to make the examination whilst the child is asleep, when we can open the eye with ease, and see it in a natural condition—I mean not congested by excitement—or if such an opportunity does not offer, I turn the child with its back, or its side to the light, and then, shading the eye with my hand, I soothe the child, and attract its attention to some object, by which means, and the exercise of a little patience, I seldom fail in attaining my object.

Ulcers assume a variety of aspects according to circumstances. The superficial laminæ only of the cornea may be implicated, or the entire substance may be penetrated. An ulcer may be perfectly clear and transparent, as if a piece had been chipped out of the cornea, or the surface may be covered with opake matter, and a cluster of vessels be seen running from the margin of the cornea to the ulcer. In this latter form the eye is irritable, there is intolerance of light, the conjunctiva is more or less congested, and the general symptoms are those of inflammation of the organ. If, however, there is neither vascularity nor irritability, and the surface of the ulcer is simply opake, we may regard it as healing, for the opacity is caused by effusion of lymph, which ultimately fills up the cavity. The formation and bursting of pustules and phlyctenulæ is one of the most frequent sources of ulcers, and I have seen some very formidable instances succeeding to small-pox. They are common too after scarlatina and measles. That peculiar inflammation of the sclerotic, called rheumatic ophthalmia, is often attended with the formation of troublesome superficial ulceration, which destroys the conjunctival covering of the cornea, and spreads extensively, but does not penetrate into the substance of that tunic.

Whatever may be the character of the ulcer, it is very desirable to put a stop to its progress, and promote its healing with the least possible delay. If it be inflammatory, the application of a few leeches will be of service, and the bowels should be freely opened ; a blister may be applied behind the ear, and relief will be afforded by frequent fomentations with warm water. When the acute symptoms have subsided, a weak solution of nitrate of silver will be the best application, beginning with one grain to the ounce of distilled water, and gradually increasing the strength to four grains. A full drop of this should be placed in the eye twice a day, by drawing down the lower lid and gently sweeping a large camel hair brush, loaded with the solution, along the fold between the lid and the globe, whence the fluid will be instantly carried over the cornea.

The treatment of the transparent ulcer should be directed to the removing, as far as possible, of that condition of the constitution from which it originates. As it usually occurs in feeble, unhealthy, pallid children, every means ought to be taken to improve the general health, by diet, salt and water ablution, friction of the skin, fresh air, and medicine. Of the latter, tonics are indicated, but we find that those which do good in some cases, are of no service in others. Three, however, are chiefly to be relied on, namely, steel, quinine, and iodide of potassium. The latter has been recommended by Mr. Turton, of Sheffield, in a recent number of the *Provincial Journal*, and I can bear ample testimony to its efficacy in strumous ophthalmia. The following is the formula which I have been in the habit of prescribing for the last four years :—

| | |
|---|---|
| ℞. Potass. iodid. | gr. ij. ad. iv. |
| Sodæ sesquicarb. | gr. iij. ad. v. |
| Aquæ destillatæ | oz. ½. M. |

The above may be taken twice or three times daily, or it may be advantageously combined with infusion of gentian, half an ounce of each being taken at a dose. This appears to exercise a highly beneficial effect upon the constitution, and will frequently succeed when steel and quinine have failed. As a local application to transparent ulcers, there is nothing equal to nitrate of silver. If possible, I touch the ulcer once a day with a strong solution, (from six to ten grains to the ounce of water,) but as some dexterity and care are requisite, I do not think it prudent to permit the parents to do this, but, (if I do not see the patient myself,) desire a weaker solution, (three or four grains to the ounce,) to be used twice daily, in the manner I have already described.

When an ulcer has completely eaten through the substance of the true cornea, its further progress is for a time resisted by the membrane of the aqueous humour, which projects forwards through the opening, in the form of a delicate vesicle. It is of considerable importance that this should be attended to, for if neglected, and the ulcer perforates that lamina, the aqueous humour will flow out, the iris fall forward, and a portion becoming entangled in the ulcer, will project through it, appearing like the head of a fly, whence the name "myocephalon" which has been applied to it. In the former case it is best to touch the vesicle with a strong solution of nitrate of silver: in the latter case it is very desirable to cause retraction of the iris, or if that cannot be accomplished, (which is too often the case,) to prevent further protrusion, which may be done by exciting the effusion of lymph, and so glueing the portion already protruded to the margin of the ulcer. The extract of belladonna should be applied freely to the brow, and the parts touched thrice a day with a solution of nitrate of silver, by which the desired effect will be produced.

I have mentioned a form of superficial ulcer attendant upon rheumatic sclerotitis. When this exists, it is accompanied by an unusual amount of irritability, and intolerance of light, and the appearance is as if a portion of the surface of the cornea had been peeled off. The weak solution of nitrate of silver is here also the best application, but it is from general treatment that most benefit is to be expected. The tongue, in such cases, is usually very foul, and the digestive organs much deranged. A dose of calomel, or calomel and Dover's powder at night, followed by a brisk aperient in the morning, should be administered, and these should be followed up by alteratives, until the tongue becomes clean. In many instances colchicum is then highly efficacious, but I have found the greatest advantage to result from the exhibition of the following:—

R. Quinæ disulphatis, gr. j. ad. gr. ij.

Sodæ sesquicarb. gr. iv.

Pulv. tragacanth. co. gr. x. M.

Ft. pulvis, ex aquæ cinnamom. uncia sexta quaque hora sumendus.

It may also be necessary to abstract a moderate quantity of blood by cupping or leaching, and to excite counter-irritation by blistering behind the ear.

*Prov. Med. and Surg. Journ.*

